# Electrical Signal Modeling in Cochlear Implants. Study of Temperature and Humidity Effects

**DOI:** 10.3390/mi12070785

**Published:** 2021-06-30

**Authors:** Maria-Alexandra Paun, Vladimir-Alexandru Paun, Viorel-Puiu Paun

**Affiliations:** 1 Department of Engineering, Swiss Federal Institute of Technology (EPFL), Route Cantonale, 1015 Lausanne, Switzerland; 2 Five Rescue Research Laboratory, 35 Quai D’Anjou, 75004 Paris, France; vladimir.alexandru.paun@ieee.org; 3 Faculty of Applied Sciences, Physics Department, University Politehnica of Bucharest, 313 Splaiul Independenței, Sector 6, 060042 Bucharest, Romania; viorel_paun2006@yahoo.com; 4 Academy of Romanian Scientists, 54 Splaiul Independentei, Sector 5, 050094 Bucharest, Romania

**Keywords:** cochlear implant, humidity, temperature, MATLAB, Simulink

## Abstract

The present paper discusses the climatic effects of humidity and temperature on cochlear implant functioning and the quality of the electrical sound signal. MATLAB Simulink simulations were prepared, offering insights into signal behavior under such climatic parameter changes. A simulation setup of the cochlear implant was developed, where a source type selection was used to change between a voice recording and a “chirp” sound. In addition, a DC blocking filter was applied to the input signal. A simulation code, with the application of the climatic influence via the air attenuation function, was developed. Thereby, the attenuation of temperature and humidity in the sound atmospheric circulation of the input signal, at T = 0 °C and RH = 0% and at T = 36 °C and RH = 40% was graphically represented. The results of the electrical pulse generator for each of the eight channels, with the IIR filter, Gaussian noise, temperature variation, humidity influence, and control of denoise block activity, were thus obtained.

## 1. Introduction

Hearing is an important component of human beings’ quality of life, at any age. It is well known that hearing capacity decreases with age or due to some professions. Hearing loss may ultimately lead to deafness. The decrease of hearing capability towards deafness, and its inherent social complications, can be a major source of discomfort. However, many babies are born or develop during early childhood (ages 1–3 years) with profound hearing impairments. As babies learn through speech recognition, the fact that they cannot hear may lead to the mispronunciation of words and difficulties in learning language. These challenges may result in major setbacks in their life and in human interaction, with respect to other children their age. Without proper treatment, they may require special assistance within society. 

Numerous solutions for resolving this issue, some of which are more or less efficient, have been proposed. Among the best solutions so far, with excellent hopes for positive development in terms of technical performances, is the cochlear implant [1,2,3,4,5,6,7,8,9]. A cochlear implant is an electronic medical device that replaces the function of the damaged inner ear by enabling radio communication between two antennas and work through electrical stimulation of an array of electrodes implanted in the cochlea. While hearing aids amplify sounds, cochlear implants perform the work of damaged parts within the inner ear (cochlea) in order to provide sound signals to the brain [10]. More precisely, a cochlear implant provides direct electrical stimulation to the auditory (hearing) nerve in the inner ear. In cochlear implants, important aspects to consider are the biocompatibility, safety, electric stimulation, signal processing, selection of optimal radio frequency, and placement of electrodes [10]. A good knowledge and use of remote powering are necessary in the development of cochlear implants [11,12,13,14,15,16,17,18,19].

The hearing aid, an external device which amplifies the signal, has a retail price between USD 1000 and USD 6000 each, and is available via companies such as Bernafon, Oticon, Phonak, Resound, Signia (formerly Siemens), Starkey, Unitron, and Widex [20]. On the other hand, the cochlear implant, which involves a medical surgical procedure, can reach costs of up to USD 50,000 and USD 100,000, and is available through MED-EL (Innsbruck, Austria), Cochlear Ltd. (Sydney, Australia), and Advanced Bionics (Santa Clarita, CA, USA).

In 2019, the global market value for hearing aids was USD 8.99 billion (with a CAGR of 8.2%) and USD 1.67 billion for cochlear implants (with a CAGR of 10.6%) [21]. The majority of hearing aids are sold in developed markets, with the highest percentage found in Europe (41%), closely followed by North America (29%), and Asia and the Pacific (21%). The lowest percentages are found in South America (7%) and Africa (2%) [22].

The objective of this paper was to study humidity and temperature effects on the quality of sound within cochlear implants via a MATLAB Simulink complete simulation approach. Both humidity and temperature sensors work together to provide a correct and complete response to the sound signal influence of climatic changes. The aim of this work is a theoretical one, of pure precise modeling, as a first step in the validation of our novelty approach. The complex validation process will be made available in a subsequent paper when we will take measurements and the experimental data will be properly analyzed and compared with the simulation data.

It is very important to investigate the quality of the environment from the point of view of the sensor response individualization and correctness of the information. In this project, we aimed, on one hand, to study and monitor the environment in the vicinity of the implant. Among the parameters of interest were temperature and humidity. On the other hand, our objective was to individualize the behavior of the cochlear implant for each patient. The latter is an important task in order to properly identify the individual treatment required by each patient. It is said that we have patients, not diseases. Every patient with this implanted device displays a different comportment with respect to individual living and working conditions. 

As the patient with the cochlear implant will have a dynamic life, including a changing environment, humidity and temperature information from their surroundings will be of crucial importance to the proper functioning of their cochlear implant. This information will be fed into the system. More specifically, the humidity sensor will help in calibrating the loudspeaker of the implant, which receives the information from the exterior and then sends it to the internal unit.

Cochlear implants (CIs) are implantable auditory prostheses designed to restore access to sound in deaf individuals via direct electrical stimulation of the auditory nerve. Each CI consists of a speech processor with a microphone worn outside the ear, which processes sounds and then transmits the processed signal via a transmitting coil to a receiver under the skin. This receiver converts the signal into electric current, which is then sent to the cochlear implant electrode array implanted in the cochlea. 

The microphone transmits processed information via the transmitter coil through the skin, to an internal receiver in the cranial bone that converts this signal to electrical pulse trains to be delivered to the electrodes implanted in the cochlea.

Cochlear implants have their own dynamics towards performance, being subject to changes month by month and always offering better, more efficient variants [23].

Used as the main component in implantable stimulators, the second-generation integrated circuit for the Active Books stimulation microsystem in neuro-prostheses, has already been realized. The microprocessor used is composed of two sensors, one temperature and one humidity [24].

The humidity sensor output varies linearly with relative humidity (RH) with a normalized sensitivity of 0.04% RH over the range of 20–90% RH. The temperature sensor has a nonlinearity of 0.4% over the range of 20–90 °C and a resolution of 0.12 °C. The stimulator is the first of its kind to include two integrated temperature and humidity sensors.

The idea, presented in this paper, of using a single temperature and humidity sensor in the IC was developed during an activity carried out at the University of Cambridge, which was published in specialized journals [25]. This avoids the use of two independent and bulky sensors, meaning it is miniaturized and adapted to a cochlear implant type prosthesis. 

The relationship between different weather variables, such as temperature and humidity, can be quite confusing. Temperature and humidity are connected either directly or indirectly. For the purpose of analyzing the results, it is of the essence to understand their mutual dependence.

Humidity is the amount of water vapor, the gaseous state of water, in the air, and it is usually invisible. The maximum amount of water vapor in the air depends on air temperature. Absolute humidity (AH) is the water content in the air, i.e., the mass of water vapor included in a particular volume of air, expressed in g/m^3^.

Temporal variation of signal strength, when exploring the results from our experiments in both summer and winter, made it evident that signal strength had both short-term (diurnal) and long-term (seasonal/weekly) variation. Interestingly, the variation was notably different in each period. Diurnal variation (day/night) was clearly apparent in summer, whereas seasonal variation was easier to detect in winter and between different seasons (summer/winter). These variations were not random, but were mainly cyclic, following a certain distinct pattern.

This paper is organized into five sections. After the introduction, the paper presents the materials and methods. In the third section, the paper presents the results, while in fourth section the obtained results are discussed. Finally, the work is concluded in the fifth section. 

## 2. Materials and Methods

This section is intended to present the materials used in setting up our analysis and problem solving. We start by giving the main scientific arguments for our work, then we present the cochlear implant and make an important analysis of the electrical signal in the cochlear implant, and its behavior based on temperature and humidity, via MATLAB Simulink simulations. Previous works analyzed temperature effects on CMOS (complementary metal oxide semiconductor) Hall sensors [26] and proposed a pulses generator and its behavior [27]. We apply in this paper a similar technique to the sound wave as the one applied to the light wavefront aberration correction via programmed computer code in paper [28].

A precise three-dimensional model of a cochlear implant, together with extensive simulations, were previously performed in Ansoft Ansys^®^ HFSS software, which is used for electro-magnetic simulations. Results were presented in papers [29,30,31], which provided insights into the proper functioning of cochlear implants with the development of a multi-layer tissue model for the transmitter and receiver antennas constituted mainly of skin, mastoid bone, and brain. The proposed model highlighted power loss attenuation of the electromagnetic sound wave while travelling between the outside and the inside antennas and emphasized the modeling of the antennas with precise dimensions and varying implantation depths. By looking at these papers, we found, amongst other simulations, the results for designed loop antennas implanted in different types of tissues (placed both in air and in a multi-tissue environment), the electric field, the antenna return loss, the Smith charts, and the radiation pattern. Phase and gain attenuations were also evaluated through S-parameter analysis. 

However, the purpose of this paper was to model the electrical signal in the cochlear implant in a simulation based on discrete components (functioning blocks) in a Simulink environment, in remote powering mode, subject to various climatic changes. At this time, we also have the pleasure to introduce the unique idea of adding, for the very first time, temperature and humidity sensors in the circuit in order to compensate for these variations that greatly affect the quality of the sound signal, with arguments being given later on in this section. These temperature and humidity sensors (here modeled as filters to the signal) will give an idea of the electrical signal behavior with these climatic changes. 

### 2.1. Main Scientific Arguments for Our Work

This project aimed to improve the wellbeing of patients with cochlear implants who, for example, work outside, participate in outdoors activities, have a fever, etc. In these situations, the external medium changes in temperature and humidity. This influences the quality of the sound signal. The quality of the external sound is affected by temperature and humidity in the following ways. Hot air is less dense than the cold air and therefore the sound travels faster through the external medium. The attenuation of sound in air is also affected by humidity. The moist air (higher relative humidity) is less dense than dry air and, by consequence, like with the higher temperature, the sound travels more easily through that medium. Dry air absorbs more acoustical energy than the moist air.

In essence, we have two media: the air from the exterior, where the sound propagates, and the internal medium, which should receive the undistorted information. Additionally, there is the synchronization of the sensors, one on the outside and the other on the inside, whose conditions do not change much. 

We are greatly interested in the effect of temperature and humidity on the correct functioning of the sensors, as well as their reliability. For example, there is an important difference between the level of humidity indoors and outdoors, as well as in different cities. The humidity and temperature also change with respect to the seasons, as well as from daytime to nighttime. There is likewise an increase in temperature in cities which have lakes (e.g., Geneva and Lausanne). The quality of sound in air does not change with altitude directly, but does so through the agency of temperature, which, once again, tends to decrease if we go higher in altitude. 

### 2.2. Cochlear Implant Description

**Figure 1:**
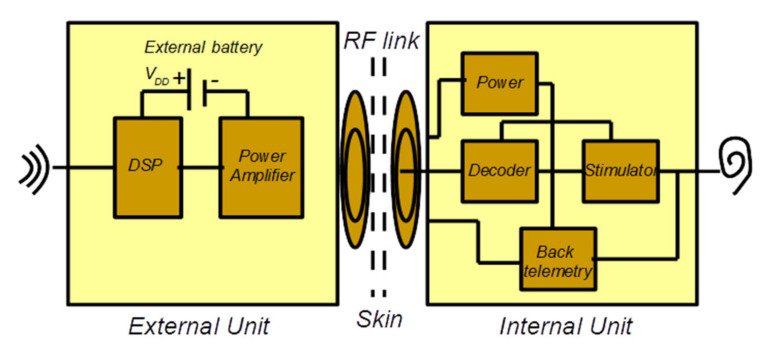
Schematic block diagram for the cochlear implant.

The schematic diagram for a cochlear implant is illustrated in Figure 1, where we can observe the main functional blocks necessary for its realization. There are two units, namely the external unit and the internal unit [10]. The first is comprised of a power amplifier, DSP (digital signal processing), and an RF transmitter. It is the DSP that is responsible for extracting the information in sound and converting it into bits to send over the RF link. On the other hand, the internal unit is composed of the RF receiver and a stimulator. However, as there is no battery in this unit, it is the job of the stimulator to firstly derive power from the RF signal [10]. When the simulator is charged, it will proceed to make a decoding of the RF bit information and convert it into electric currents for the electrodes. Additionally, there is a feedback loop with the objective of accounting for the critical electrical and neural signals within the implant and sending them back to the external unit.

**Figure 2:**
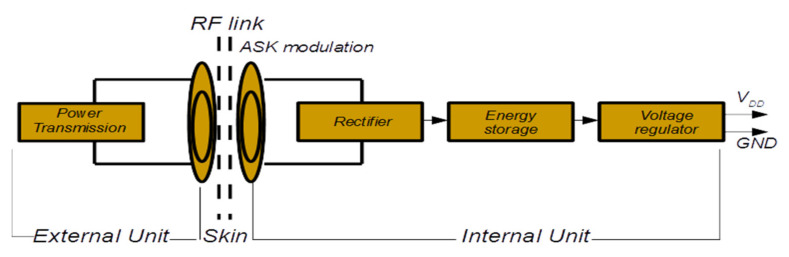
Basic architecture of the cochlear implant.

Figure 2 presents the basic architecture of the cochlear implant [18], emphasizing the power transmission, rectifier, energy storage, and voltage regulator. The RF link is employed for both the DC power supply voltage of the cochlear implant and the data transmission using the modulation technique. The system can, for example, use ASK modulation, which is commonly used for data transmission and power in biomedical implants. The DC power supply voltage of the implant consists of the rectifier converting the RF carrier into a useful DC supply voltage, an energy storage element and voltage regulator [18]. The data transfer occurs at the radio frequency level between transmitter and receiver antennas. The maximum stimulation rate is influenced by the bit rate and the frame rate in the RF transmission link. The bit rates vary from 250 kbits/s to 500 kbits/s in different systems [10]. It is worth mentioning that the internal unit is positioned inside the skull, more precisely in the mastoid bone.

Some of the design goals of intracochlear electrodes are the insertion depth, coupling efficiency, and insertion trauma [10]. It is known that reducing the electrode-to-nerve distance decreases power consumption and interaction between channels. In addition to this, it is crucial to have wireless communication, to reduce possible infections and increase patient comfort. In order to be able to extend the battery life of the external unit and relax the RF transmission efficiency, one must master the low power design [10]. In paper [32], a total power consumption less than 1 mW was reported for the internal unit. Most current systems use discrete components to achieve the back telemetry power supply.

### 2.3. Humidity and Temperature Effects on the Quality of the Sound Wave and Signal Strength

Herein, we will address issues such as the effect of temperature and humidity on the speed of sound associated to the quality of the signal sound, therefore understanding the impact on the reception of sound in the microphone of the cochlear implant, attenuation of the signal, and the decrease of sound quality.

As we know, the speed of sound is affected by temperature and humidity; because it is less dense, sound passes through hot air faster than it passes through cold air. For this reason, temperature gradients cause refraction effects. Moreover, as sound propagates through the air, the air absorbs energy from the sound wave, attenuating (weakening) it. The effect is significant only at frequencies greater than 2 kHz, and increases with frequency. The attenuation of the sound in the air is affected by the relative humidity. Dry air absorbs far more acoustical energy than moist air. This is because moist air is less dense than dry air (water vapor weighs less than air) [33]. 

The speed of sound in air varies depending on several factors, including humidity [34], and is determined by the air itself and is not dependent upon the amplitude, frequency, or wavelength of the sound. For an ideal gas, the speed of sound depends only on its temperature and is independent of gas pressure. This dependence also applies well to air, in good approximation, and can be regarded as an ideal gas. Environmental effects change the speed of sound and the absorption of sound in air. Even seemingly small percentage changes may cause serious listening problems in enclosed acoustic spaces [35]. 

Temperature will have an effect on microphone performance. Sensitivity levels can be directly affected by extreme environmental conditions. As the temperature approaches the maximum specifications of the microphone, its sensitivity specification will decrease. The owner will need to be aware of not only the operating temperature, but also the storage temperature of the microphones. If operated or stored in extreme conditions, the microphone can be adversely affected and will also require calibration more often [36].

It is worth noting that the speed of sound changes with temperature, slightly with humidity, but not with air pressure (atmospheric pressure). Thus, it is only because of the decreasing air temperature, which decreases with altitude, that the speed of sound decreases.

Changes in weather conditions affect radio signal strength. In terms of signal strength variation, temperature as a variable has a negative, linear effect on signal strength in general, while high relative humidity may have some effect, particularly when temperature is below 0 °C. The effect of weather conditions on response quality (e.g., signal strength) were explored in several studies. The related variables used to measure radio signal strength, temperature, and humidity, were defined in [37]. Some studies report that temperature was the dominating factor affecting signal strength [37], while others claimed that humidity was the main reason [38,39]. Thus, it is essential to explore the factors affecting radio waves link quality in order to mitigate the impact of temperature and humidity variations and to adapt to varying conditions [40].

The relationship between different weather variables, such as temperature and humidity, can be quite confusing. Temperature and humidity are connected to each other either directly or indirectly. For the purpose of analyzing the study results, it is of the essence to understand their mutual dependence. Absolute humidity *AH* (g/m^3^) can be defined, e.g., as a function of temperature and relative humidity as [41]:

(1) $$AH\left(t,RH\right)=216.7\left[\frac{\frac{RH}{100\%}Aexp\left(\frac{mt}{Tn+t}\right)}{273.15+t}\right]$$

where *t* (°C) is the actual temperature, *RH* the actual relative humidity (%), *m* = 17.62, *Tn* = 243.12 °C, and *A* = 6.112 h Pa.

It is the basic formula used in the module for correcting atmospheric attenuation of sound, when traversing space through the air, from the emitting source to the patient’s ear. Equation (1) has the role of calculating *AH* at various temperatures to be used as the input data in the module for adapting the frequency and the sound power, in order to obtain the necessary corrections for a normal standard sound.

Formula validity. The temperature must be greater or equal to −40 °C, and less or equal to 100 °C. Several values for absolute humidity, *AH*, were calculated according to the above formula. The *AH* values which were obtained are *AH* (−40 °C, 10%) = 0.02 g/m^3^, *AH* (0 °C, 20%) = 0.97 g/m^3^, *AH* (20 °C, 60%) = 10.37 g/m^3^, and *AH* (20 °C, 85%) = 14.7 g/m^3^.

### 2.4. Simulation of Cochlear Implant Using MATLAB Simulink

In this section, the simulation of the cochlear implant using MATLAB Simulink is detailed, including system blocks, block parameters, some associated code, and the model hierarchy used to evaluate the electrical behavior of the cochlear implant. This tool allows us to rapidly and correctly investigate the device under discussion, as well as to assess the quality of the electrical radio signals.

This paper discusses, for the first time in literature, the effect of humidity and temperature on the quality of processed sound. The calibration of sound was carried out on the real temperature and on the existing humidity in the sound emission space. Prior to this, these considerations were not taken into account when designing cochlear implants.

The responses of the sensors to the change of temperature (temperature effect) and to the change of the environmental conditions regarding the humidity (effect of humidity) were highlighted by numerical simulation. These evidently marked the alteration of sound quality depending on the change of environmental conditions, namely the change of temperature and ambient humidity.

The designed and developed conceptual blocks, such as the cochlear implant signal source block and cochlear implant signal from Workspace block, were both represented in MATLAB Simulink. The first concept, made to work like source type selection, was used to change between a voice recording and a “chirp” sound, and a DC blocking filter was applied at the input signal.

In addition, the cochlear implant filter bank block was represented in the MATLAB Simulink so that signal processing of the input source would use two filter types: FIR (finite impulse response) and IIR (infinite impulse response). To increase the degree of confidentiality, the FIR filter was applied in a cascade to the eight frequency bands.

Finally, the denoise stage block was schematically represented, together with stimulation pulse generator block.

#### 2.4.1. Simulation Setup

In the standard cochlear implant, an adequate frequency range can be set from 70–350 Hz up to 3500–8500 Hz, but as far as limits are considered 75 Hz is the lower limit and 8500 Hz is the upper limit. However, it is necessary to find the best frequency range for CI speech perception, thus, we found the best frequencies in the range considered optimal for very clear language transmission. Making an analysis almost exhaustive based on several frequencies in the simulations demonstrates that comb filtered speech (spectrally deprived speech) can be used in the modeling of CI speech perception. Thus, we carried out this study of comb filtered speech recognition in eight frequency ranges, chosen as reference intervals for the whole process. 

##### Simulink Presentation

Here, we briefly present Simulink software, including what it is in principle and what it can be used for. In general, engineers and scientists use Simulink^®^ to perform multidomain modeling and simulation, because it can reuse models across environments to simulate how all parts of the system work together. With Simulink, we can do the following:

- Model our system across domains using specific tools and prebuilt blocks;
- Develop large-scale models through componentization with reusable system components and libraries;
- Combine our models into one system-level simulation even if they were not built in Simulink;
- Run massive simulations in parallel on a multicore desktop, computer cluster, or the cloud, without writing large amounts of code;
- Share simulations as standalone executables, web apps, and Functional Mockup Units (FMUs).

Industry-specific applications can also be designed in Simulink using prebuilt blocks, so we do not have to create them ourselves. For example, aircraft propulsion systems can be designed with Aerospace Blockset™, or audio and video systems with Signal Processing tools, as in our case. As for the math behind the application Simulink, we will refer to the simulation applications for making graphics. 

Standalone executables can be complete simulation apps that use MATLAB graphics and UIs designed with the MATLAB App Designer. To provide browser-based access to a deployed simulation, it will create a web app and host it with the MATLAB Web App Server™. Standalone Functional Mockup Units (FMU) are binaries that adhere to the Functional Mockup Interface (FMI) standard and can be used in an external simulation environment.

A standalone executable is a program that does not depend an Objective Caml installation to run. This facilitates the distribution of binary applications and robustness against runtime library changes across Objective Caml versions. 

The Objective Caml native compiler produces standalone executables by default. Without the custom option, however, the byte code compiler produces an executable which requires the byte code interpreter *ocamlrun*. For example, imagine the file example.ml is as follows: 

Let f (x) = x + 1;

Print_int (f, 18);

Print_newline (…);

Then the following command produces the (approximately 8 k) file example.exe: 

Ocamlc -o example.exe example.ml

This file can be executed by the Objective Caml bytecode interpreter: 

$ ocamlrun example.exe

These are some of the subroutines defined as mathematical applications in the software used to run programs in Simulink.

#### 2.4.2. Signal Processing Strategy

The purpose of the filter bank signal processing block was to decompose the input speech signal into eight overlapping subbands. There is more information contained in the lower frequencies of speech signals than in the higher frequencies. To get as much resolution as possible where the most information is contained, the subbands were spaced in such a way that the lower-frequency bands were narrower than the higher-frequency bands. 

##### Noise Adaptive CI System

The advanced CI system is capable of detecting a change in the background noise with no user intervention and changes the noise suppression parameters to previously determined (during training) optimal parameters for that particular background noise. A block diagram with a first-order low-pass filter, which is used at the end to smooth out any fluctuations, was proposed.

Let us consider an additive noise scenario, with clean, noise, and noisy received signals represented by *x_m_*(*n*), *d_m_*(*n*), and *y_m_*(*n*), respectively, where m denotes the window number. A priori and a posteriori SNRs (signal-to-noise ratio) for the speech spectral estimation are given as follows:

(2) $$y_{m}\left(n\right)=x_{m}\left(n\right)+d_{m}\left(n\right)$$

(3) $$Y_{m}\left(k\right)=X_{m}\left(k\right)+D_{m}\left(k\right)$$

(4) $$\xi_{m}\left(k\right)=\frac{\lambda_{x}\left(k\right)}{\lambda_{d}\left(k\right)},\;\gamma_{m}\left(k\right)=\frac{Y_{m}^{2}\left(k\right)}{\lambda_{d}\left(k\right)}$$

where *ξ_m_*(*k*) denotes the a priori SNR, *γ_m_*(*k*) denotes a posteriori SNR at the frequency bin *k*, *λ_d_* denotes the noise variance, and *λ_x_* denotes the clean speech variance. Frequency bin is a discretized frequency, which transforms continuous frequency into a discrete one. A priori SNR and a posteriori SNRs are obtained by using the “decision-directed” approach as:

(5) $$\xi_{m}\left(k\right)=\alpha_{dd}\frac{X_{m-1}^{2}}{\lambda_{d}\left(k\right)}+\left(1-\alpha_{dd}\right)max\left(\left(\frac{Y_{m}^{2}\left(k\right)}{\lambda_{d}\left(k\right)}-1\right),\;\xi_{min}\right)$$

(6) $$Y_{m}\left(k\right)=\frac{Y_{m}^{2}\left(k\right)}{\lambda_{d}\left(k\right)}$$

where *α_dd_* is a smoothing parameter [42,43], and *ξ_min_* is a small number greater than 0.

SNR is defined as the ratio of signal power to the power of background noise. According to [42,43], the use of the non-ideal a priori SNR estimate, which was derived using the speech spectral estimation of the previous window, led to erroneous spectral estimates. This error gets fed back into the system. To minimize this error, a modified a priori SNR estimate, based on the previous noisy speech spectra (rather than enhanced spectra), was used. 

##### Methods

In the present study, we used eight bands for the eight frequency ranges, from the lower limit of 70 Hz to the upper limit of 8500 Hz. The eight frequency ranges chosen were the following: 70–250 Hz, 70–350 Hz, 350–6500 Hz, 250–6500 Hz, 70–6500 Hz, 350–8500 Hz, 250–8500 Hz, and 70–8500 Hz. We used the model of 8-channel implants. 

##### Results and Discussion

The best results of comb filtered words frequency were recognized in the frequency range of 250–6500 Hz, with minimal recognition in the broadest frequency range.

Since the discovery of multi-channel devices, research has demonstrated that hearing performance improves with increasing numbers of spectral channels [44,45,46]. However, the benefits are highest when advancing from 1 to 8 channels, with most of them being on 5 channels. While current CIs contain between 12 and 24 electrodes, previous studies showed that increasing the number of stimulation sites beyond 8 yielded no additional gains in speech understanding [47,48,49].

At equidistance on the basilar membrane, distribution spectral bands, in accordance with normal tonotopical organization of the cochlea [15], was chosen by analogy with equal distances between the electrodes in the chain of the implant, i.e., the speech bands were arranged around the central frequencies of the five-channel implants, operating in four different frequency ranges (see Table 1).

The cochlear implant block representation was effectuated in MATLAB Simulink, and contained an input source, cochlear speech processor, filter bank signal processing with designer filter bank, FIR and IIR filter bank response, an optional denoise stage, and electrical pulses with a pulse generation by 8 channels.

Thereby, the cochlear implant signal source block, (represented in MATLAB Simulink) was introduced, where source type selection was used to change between a voice recording and a “chirp” sound, and again on the DC blocking filter, which was applied to the input signal. Evidently, a major focus on detailing software was associated with the block structure of the input signal source, when the FIR filter was applied in cascade to the 8 frequency bands (arranged on the 8 channels, one on each channel).

In the following, the most important functional blocks of the electrical model of the cochlear implant will be described. Among the most relevant was the cochlear implant signal from the Workspace block representation in MATLAB Simulink. In this description we will discuss the sampling of the input signal, which was done at a frequency of sampling set at the fixed value of fs = 22,050 Hz.

Two more of the modules used included the cascade FIR bank block representation, with three successive cascaded high band/low band blocks, and the IIR filter bank block representation, with the IIR filter bank (with 8 outputs), the rectifier (with 8 inputs), and the supplementary block of 8 biquads applications.

## 3. Results

This section presents the simulation results and interprets them.

### 3.1. Filter Bank Frequency Specifications

The filter bank frequency specifications, with normalized band edges in the (−1,1) interval, were as follows: channel 0: (0,0.04); channel 1: (0.04,0.08); channel 2: (0.08,0.12); and channel 3: (0.12,0.16). In this example, the four low-frequency bands were equally spaced, while each of the four remaining high-frequency bands was twice the bandwidth of its lower-frequency neighbor. The filter bank frequency specifications, with normalized band edges in the (−1,1) interval with twice the bandwidth, were as follows: channel 4: (0.16,0.24); channel 5: (0.24,0.32); channel 6: (0.32,0.48); and channel 7: (0.48,1.0). 

**Figure 3:**
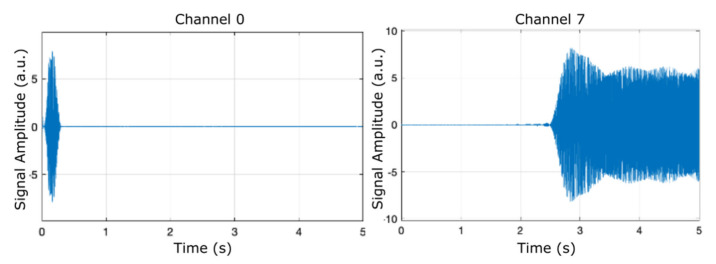
Separation of two filter banks (channel 0 and channel 7) can be observed by running the model and selecting the chirp input source.

In order to examine the frequency contents of the eight filter banks, we ran the model using the chirp source type in the input source block, as shown in Figure 3, which presents the separation of two of the eight filter banks (channel 0 and channel 7) plotted against a time frame of up to 5 s.

**Figure 4:**
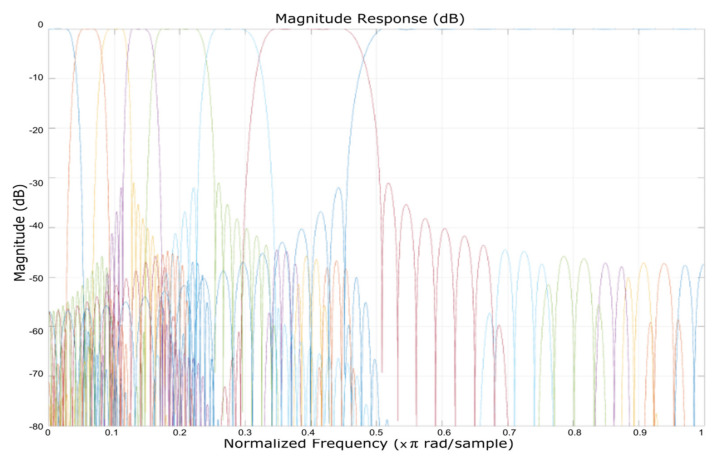
Magnitude response of the eight FIR filtered banks, which also displays the banks separation (the filter was applied in a cascade to each frequency bank).

Further on, we applied the FIR filter. Figure 4 presents the magnitude response in dB (on the OY axis) of the eight FIR filtered banks, plotted against the normalized frequency multiplied by π rad/sample (on the OX axis) that also displayed the banks separation (the filter was applied in a cascade to each frequency bank). The obtained magnitude spanned from −80 to 0 dB.

In the following, we selected the speech input source for the simulation and ran the MATLAB simulation with various parameters, selecting from a range of filters, noise sources, and denoise functions. 

**Figure 5:**
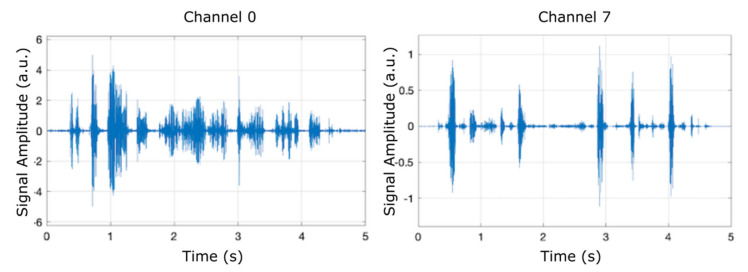
Results of electrical pulse generator for two channels (channel 0 and channel 7) under the FIR filter, without noise, and without denoise blocks (scenario as in Table 2).

**Figure 6:**
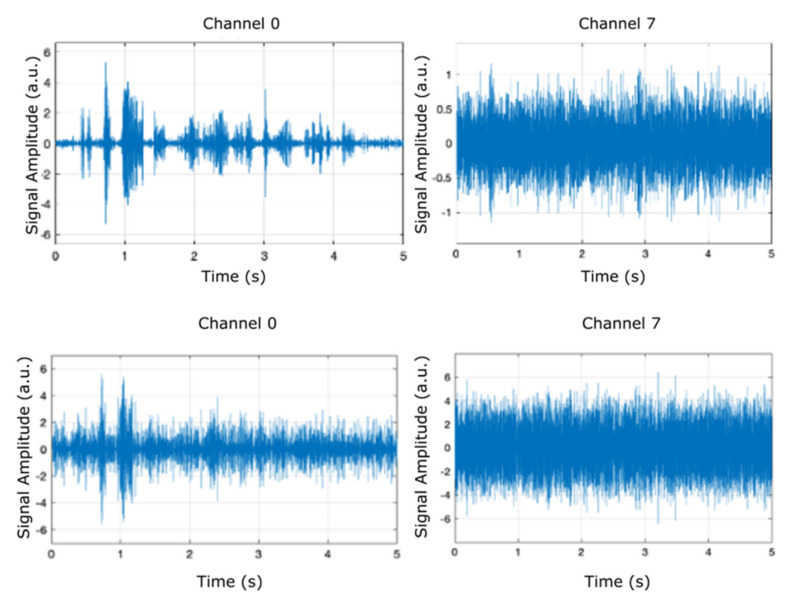
Results of electrical pulse generator for each two channels (channel 0 and channel 7) under the FIR filter, with Gaussian noise to the left, uniform noise applied to the right, and without the denoise block (scenario as in Table 3).

**Figure 7:**
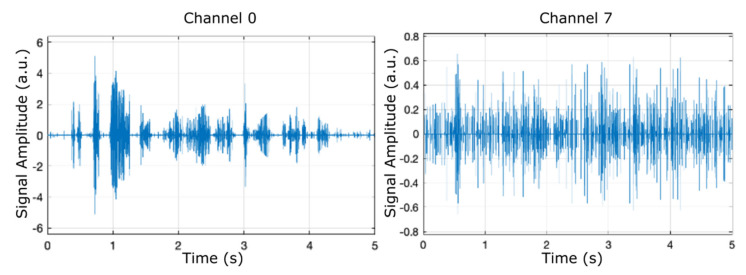
Results of electrical pulse generator for two of the 8 channels (channel 0 and channel 7) under the FIR filter, with Gaussian noise, and the denoise block active (scenario as in Table 4).

Figure 5, Figure 6 and Figure 7 display the results (signal amplitude against time) obtained corresponding to the scenarios presented in Table 2, Table 3 and Table 4, respectively, according to precise parameters (input source, filter, noise, and denoise) selection. Figure 5 shows the results of electrical pulse generator for two of the 8 channels (channel 0 and channel 7) under the FIR filter, without noise, and without denoise blocks (scenario as in Table 2), in a time interval of 5 s. Figure 6 is the result of electrical pulse generator for two of the 8 channels (channel 0 and channel 7) under the FIR filter, with Gaussian noise to the left, and uniform noise applied to the right, and without the denoise block (scenario as in Table 3), in a time interval of 5 s. Figure 7 presents the result of electrical pulse generator for two of the 8 channels (channel 0 and channel 7) under the FIR filter, with Gaussian noise, and the denoise block active (scenario as in Table 4), in a time interval of 5 s. Similar to Figure 3, in Figure 5, Figure 6 and Figure 7, out of the eight filter banks available, we have chosen to present two significant channels, namely the first and last channels (channel 0 and channel 7).

### 3.2. Influence of Humidity and Temperature on the Simulations with Addition of Temperature and Humidity Sensors

**Figure 8:**
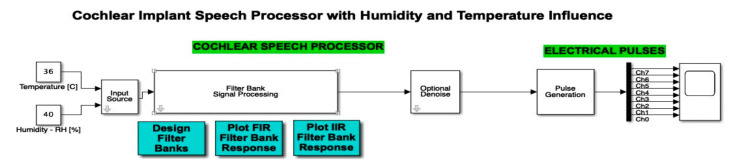
Simulink integration of the temperature and humidity. Two constants are used in order to compute the atmospheric attenuation function.

This section highlights the novelty of the investigation of climatic parameters (humidity and temperature) through the addition of such sensors in the discrete components circuit simulation. The sensors were simulated using constants integrated into the simulation environment, as can be seen in Figure 8. These sensors, present in the left-hand side of the schematic block, were included in the input source block.

**Figure 9:**
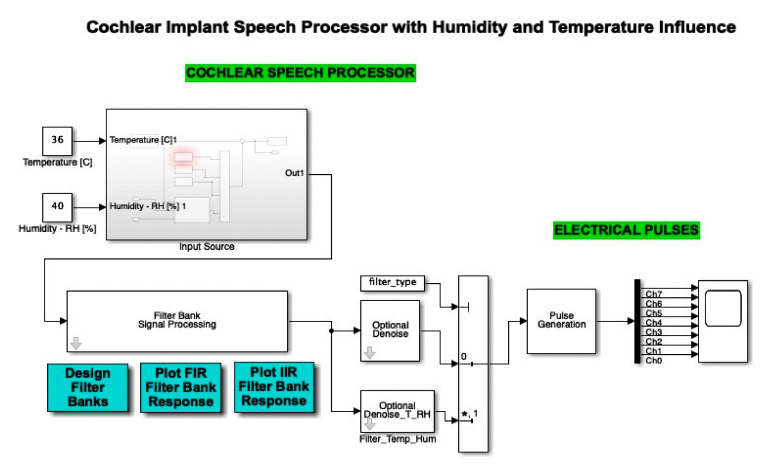
Simulink diagram of the modified input source block in the presence of the temperature and humidity influence. (*: value at the address of variable).

The atmospheric attenuation of sound was computed for the input source and applied for different temperature and humidity values. For example, Figure 8 illustrates a selection of a chosen temperature (*T* = 36 °C) and relative humidity (*RH* = 40%). Further on, in Figure 9, the input source was modified according to the read temperature and humidity. The influence was computed in the temperature and humidity effects block. In this figure, we can also observe the addition of the filter type and optional denoise stage block for both temperature and humidity parameters.

**Figure 10:**
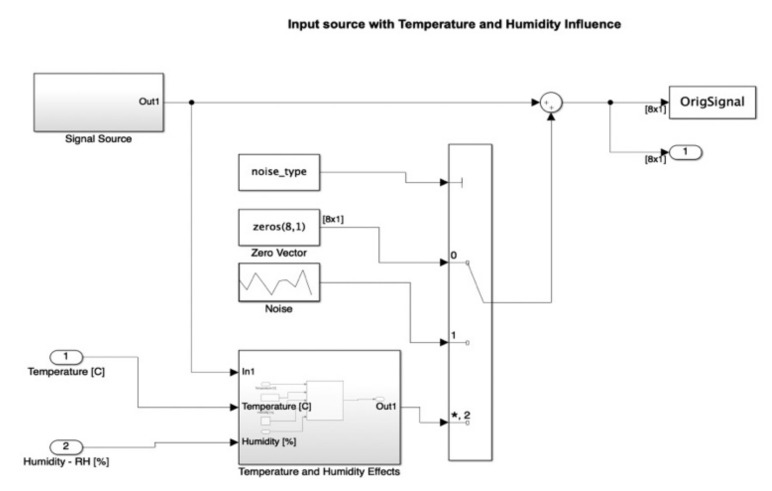
Detail of the modified input source diagram showing the temperature and humidity effects blocks added in the multiplication stage as noise. (*: value at the address of variable).

**Figure 11:**
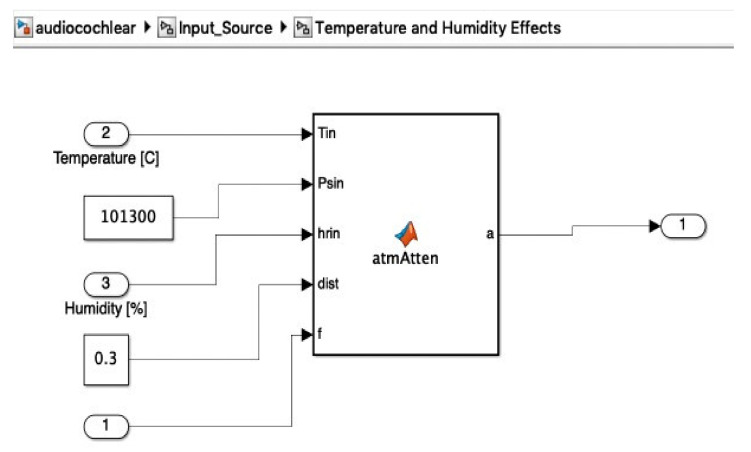
Detail of the temperature and humidity effects block, which uses the atmospheric attenuation function.

Details of the modified input source diagram, showing the temperature and humidity effects blocks, which were added in the multiplication stage as noise, are included in Figure 10. The temperature and humidity effects were applied as perturbation to the original signal, as in Figure 11.

**Figure 12:**
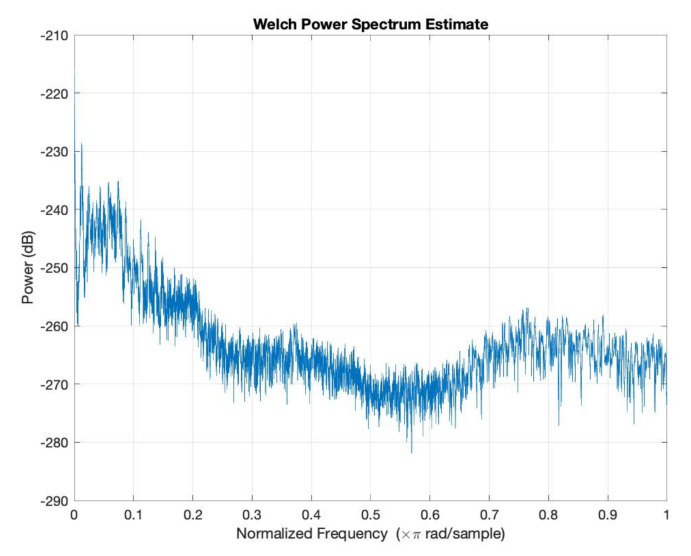
Attenuation of temperature and humidity of the input signal at *T* = 0 °C and *RH* = 0%.

**Figure 13:**
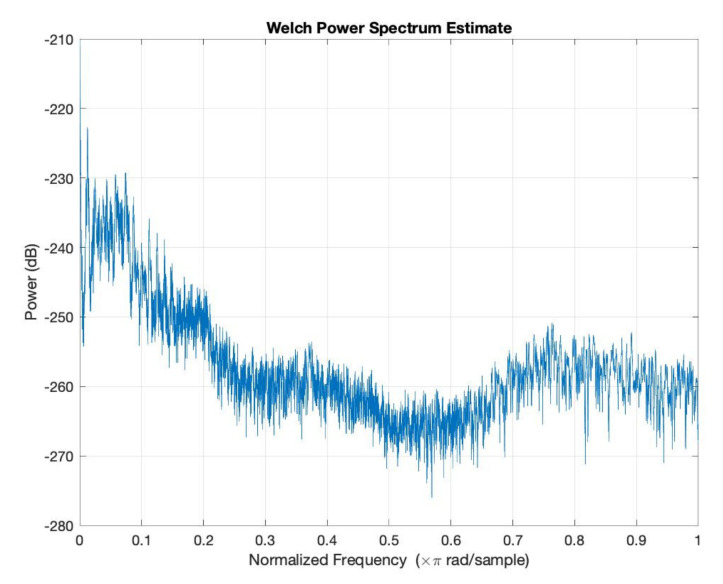
Attenuation of temperature and humidity of the input signal at *T* = 36 °C and *RH* = 40%.

In order to simulate the influence of humidity and temperature, an atmospheric attenuation of sound model was used. Figure 12 presents the attenuation of temperature and humidity of the input signal at *T* = 0 °C and *RH* = 0%. Further, Figure 13 presents the attenuation of temperature and humidity of the input signal at *T* = 36 °C and *RH* = 40%. 

**Figure 14:**
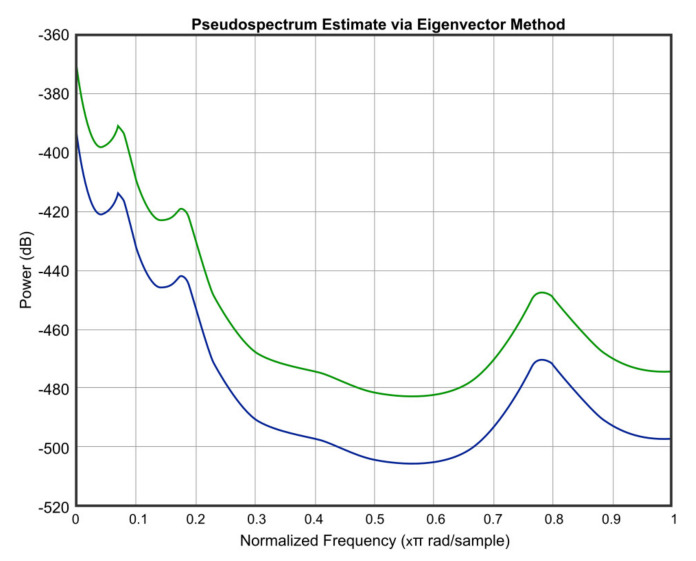
Signal behavior at *T* = 0 °C and *RH* = 0% (blue) compared to *T* = 20 °C and *RH* = 20% (green).

**Figure 15:**
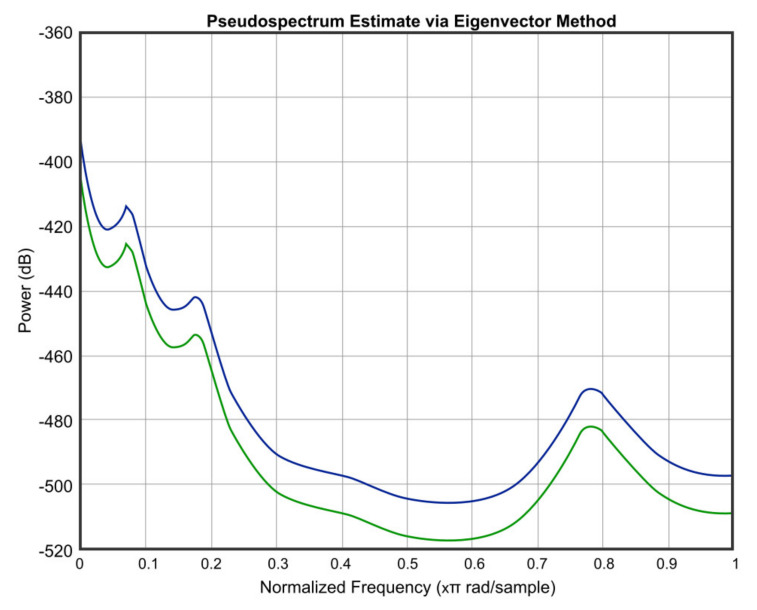
Signal behavior at *T* = 0 °C and *RH* = 0% (blue) compared to *T* = −20 °C and *RH* = 60% (green).

**Figure 16:**
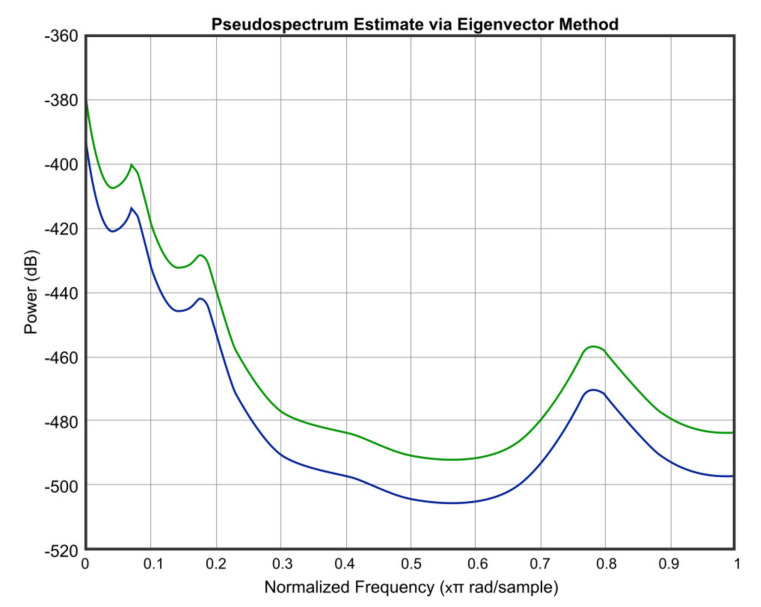
Signal behavior at *T* = 0 °C and *RH* = 0% (blue) compared to *T* = 36 °C and *RH* = 40% (green).

Figure 12, Figure 13, Figure 14, Figure 15 and Figure 16 offer the core of the novel results of our work, in the sense that we can observe quantitatively the effect of temperature and humidity on the quality of the sound. These figures present the power in dB (on the OY axis) over the normalized frequency multiplied by π rad/sample (on the OX axis). We can see that the signal suffers an attenuation (lower power) when going from *T* = 36 °C and *RH* = 40% to *T* = 0 °C and *RH* = 0%. A more detailed analysis of these results will be carried out in the dedicated discussion section.

In continuation, we present three figures, which were drawn to show the signal behavior at *T* = 0 °C and *RH* = 0%, compared to three different temperatures and humidities, namely *T* = 20 °C and *RH* = 20% (Figure 14), *T* = −20 °C and *RH* = 60% (Figure 15), and *T* = 36 °C and *RH* = 40% (Figure 16). The blue color represents the values of *T* = 0 °C and *RH* = 0%, and the green color is for the other chosen values.

The quantitative indicators used for estimation of quality parametric correlation in radio waves propagation and processing are RSSI (received signal strength indicator), PRR (packet reception ratio), SNR (signal-to-noise ratio), and LQI (link quality indicator).

**Figure 17:**
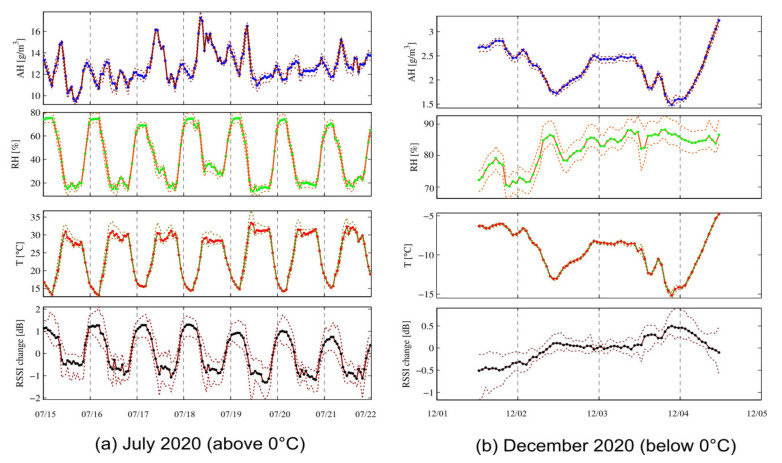
Representation of RSSI change, temperature, RH and AH in two representative months, July and December, in the year 2020. (**a**) July 2020 (above 0 °C) and (**b**) December 2020 (below 0 °C).

In Figure 17, RSSI change in dB, a temperature of 0 °C, relative humidity %, and the absolute humidity in g/m3 for two months, July (above 0 °C) and December (below 0 °C), is shown.

### 3.3. Denoise Stage of Temperature and Humidity Influence

**Figure 18:**
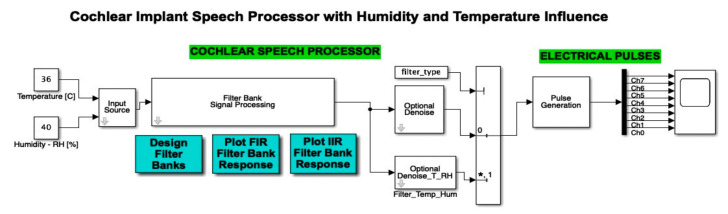
Simulink diagram of the denoise stage for the temperature and humidity influence. (*: value at the address of variable).

An additional correction step can be applied using a denoise function that corrects the temperature and humidity influence on the system, as in Figure 18.

At the beginning of the processing chain, where there is an external microphone, the sound waves arrive already affected by their way through the air and the distance from the source to the ear, which will then have to be processed electronically to reach the optimal standard values of the cochlear implant type used.

**Figure 19:**
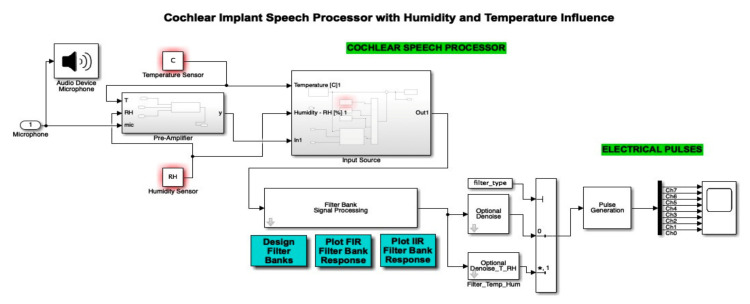
Simulink diagram of the complete simulation featuring the microphone input, the temperature and humidity sensor, as well as the additional filters and denoise blocks. (*: value at the address of variable)Finally, the result of electrical pulse generator for two channels (channel 0 and channel 7) with IIR filter, Gaussian noise, temperature and humidity influence, and the denoise block active is offered in Figure 20, which is plotted for a time interval up to 5 s. All 8 channels have an output in the range (−6,6). Similar to Figure 5 and Figure 7, Figure 8 and Figure 9, in Figure 20, out of the eight filter banks available, we chose to present two significant channels, namely the first and last channels (channel 0 and channel 7).

A Simulink diagram of the complete simulation featuring the microphone input, the temperature and humidity sensor, as well as the additional filters and denoise blocks, is displayed in Figure 19. In this way, we can see how the cochlear speech processor module was optimized by the authors through the addition of temperature and humidity sensors. The audio device microphone present in this schematic block faithfully models the microphone used in such implants in order to pick up external sound and further feed it into the system. 

## 4. Discussion

The simulation of a cochlear implant using MATLAB Simulink started by organizing a simulation setup of the cochlear implant, where a source type selection was used to change between a voice recording and a “chirp” sound. In addition, a DC blocking filter to the input signal was applied.

In a natural sequence, simulation stages were presented in detail, among which the most important ones included the cochlear implant signal source block representation in MATLAB Simulink, the denoise stage block representation, and, finally, the stimulation pulse generator block. Thus, source type selection was used to change between a voice recording and a “chirp” sound, while a DC blocking filter was applied to the input signal. Special attention was paid to simulation code with the application of the temperature and humidity influence via the air attenuation function. 

The atmospheric attenuation of sound was computed for the input source and applied for different temperature and humidity values. Moreover, the attenuation of temperature and humidity of the input signal at *T* = 0 °C and *RH* = 0% and at *T* = 36 °C and *RH* = 40% was graphically represented. 

Propagation of sound close to the ground outdoors (at levels of 1–2 m) involves geometric spreading, air absorption, and interaction with the ground. The principle effect is owed to air absorption, which is manifested by proportion of sound energy that is converted to heat as a sound wave travels through the air.

The resulting air absorption becomes significant at high frequencies and at long range, so air acts as a low-pass filter at long range. For a plane wave, the pressure *p* at distance *x* from a position where the pressure is *p_0_* is given by *p = p_0_exp*(*−αx*/2). In previous formulas, *p* was the pressure at temperature *T* (absolute temperature of the atmosphere in degrees Kelvin), and *p_0_* was the reference atmospheric pressure (1 atm = 1.01325 × 10^5^ Pa). The attenuation coefficient α, for air absorption, is dependent on frequency, humidity, temperature, and pressure.

Absolute humidity (AH) was an important factor in the diurnal variation and usually peaked in the afternoon. The diurnal variations were greatest in the summer (from 30% to 70%). It should be noted, that the use of (arithmetic) mean values of atmospheric absorption may lead to overestimates of attenuation when attempting to establish worst-case exposures for the purposes of environmental noise impact assessment.

The atmospheric attenuation of sound was computed for the input source and applied for different temperature and humidity values. Moreover, the attenuation of temperature and humidity of the input signal at *T* = 0 °C and *RH* = 0% and at *T* = 36 °C and *RH* = 40% was graphically represented. 

These values of temperature and humidity were used because *T* = 36 °C is the body temperature and *RH* = 40% is the average humidity of concert halls and of air-conditioned rooms. At the same time, they are considered as the average for reference values.

As an important observation, in the power graphs, as a function of normalized frequency (Power (dB) versus Normalized Frequency), power decreased rapidly until to a frequency of 0.6 (×π rad/sample), followed by a somewhat significant increase around a frequency value of 0.8 (×π rad/sample).

Worth mentioning are also the results of the electrical pulse generator for each of the 8 channels with the IIR filter, Gaussian noise, temperature and humidity influence, and the denoise block active.

Following the comparison, we can consider that for the first 4 channels (channels 0–3) we had a maximum fluctuation around the 1 s value (marked on the abscissa), and for the last 4 channels (channels 4–7) we had massive fluctuations for at least three values of time measured on the abscissa (horizontal axis), as happened in channel 6 for the values 1.7 s, 2.9 s, and 4 s.

## 5. Conclusions and Future Directions

To obtain the results in this project, a thorough understanding of MATLAB Simulink software was necessary. MATLAB Simulink was used to characterize the electrical behavior of the cochlear implant and to assess the audio signal and quantify the atmospheric perturbation impact on the signal quality. 

A simulation setup of the cochlear implant, where a source type selection was used to change between a voice recording and a “chirp” sound, was used. In addition, a DC blocking filter was applied to the input signal. Taking the initial intake data into account, the cochlear implant signal from the Workspace block representation in MATLAB Simulink, when the input sound was sampled, was included.

A simulation code with the application of the climatic influence, via the air attenuation function, was developed. Thereby, the attenuation of temperature and humidity in the sound atmospheric circulation of the input signal at *T* = 0 °C and *RH* = 0% and at *T* = 36 °C and *RH* = 40% was graphically represented. It can be easily seen that in the power graphs as a function of normalized frequency (Power (dB) versus Normalized Frequency), the power decreased rapidly until to frequency 0.6 (×π rad/sample), followed by a somewhat significant increase around the frequency value of 0.8 (×π rad/sample). Filter bank signal processing block was introduced, the role of which was to decompose the input speech signal into eight overlapping subbands.

The results of the electrical pulse generator for each of the 8 channels with an IIR filter, Gaussian noise, temperature variation, humidity influence, and by control of denoise block activity were thus obtained accurately. For the first 4 channels, we had a maximum fluctuation around 1 s and for the last 4 channels we had massive fluctuations for at least three values of time. For the sake of succinct presentation and article volume limitation, we have selected to present only two channels (channel 0 and channel 7) out of the eight. 

It can be said now that the novelty consists in the very idea of adding these sensors to a cochlear implant and that we obtained a modified signal, which can then be considered for future compensation. Electronic sound processing to change the parameters affected by air travel, from the sound source to the patient’s ear, is a technical novelty, as shown in this article. In the present, it was accepted and mastered in the serial production of cochlear implants by important players from the hearing aids industry (e.g., Sonova and Phonak), which now are announcing massive efforts in the future of the development of new cochlear implants.

As for future directions, the effective production of the circuit with discrete components and its subsequent testing should be carried out. 

## Figures and Tables

**Figure 20:**
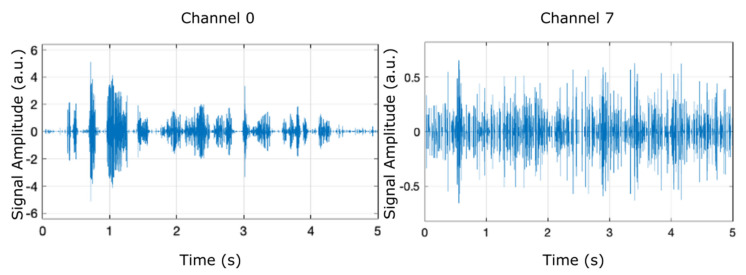
Result of electrical pulse generator for two of the 8 channels (channel 0 and channel 7) with IIR filter, Gaussian noise, temperature and humidity influence, and the denoise block active.

**Table 1:** Selection of frequencies.

Frequency Range (Hz–Hz)	Frequency 1 (Hz)	Frequency 2 (Hz)	Frequency 3 (Hz)	Frequency 4 (Hz)	Frequency 5 (Hz)
350–6500	398	781	1573	3028	5798
250–6500	289	640	1395	2860	5728
250–8500	293	693	1607	3496	7418
70–8500	120	448	1251	3108	7410

**Table 2:** Simulation parameters of scenario 1.

Input Source	Speech
Filter	FIR
Noise	NO
Denoise	NO

**Table 3:** Simulation parameters of scenario 2.

	Left	Right
Input Source	Speech	Speech
Filter	FIR	FIR
Noise	YES	YES
Denoise	NO	NO
Type	Gaussian	Uniform

**Table 4:** Simulation parameters of scenario 3.

Input Source	Speech
Filter	FIR
Noise	YES
Denoise	YES

## Data Availability

The data used to support the findings of this study cannot be accessed due to commercial confidentiality.
